# The Cohesion of Gold

**Published:** 1902-02-15

**Authors:** C. A. Hawley

**Affiliations:** Columbus, Ohio


### THE COHESION OF GOLD.

BY C. A. HAWLEY, D.D.S., COLUMBUS, OHIO.

(Read before the Ohio State Dental Society, December 4, 1901.)

In view of the fact that so many failures have been made in the making of gold fillings, either through use of different kinds of golds, or pluggers, or improper preparation of cavities, I have attempted by a series of experiments to obtain some definite knowledge of the value of different instruments for packing gold. I had a steel cavity block made similar to the one used by Dr. Black in his experiments on amalgam. I made several series of fillings. In the first two series I attempted to test the value of the three different forms of gold, pellets, ropes and tapes. In the first series twenty-four fillings were made, using three different kinds of pluggers, sharply serrated, finely serrated and smooth. The points were rectangular, 1-32 x 1-64 of an inch and all exactly the same size. Snow's automatic mallet was used, being kept at the same force for each blow, and a screw governed the stroke which was so marked that any slipping could be detected at once. The blow in this series was made light and fillings were small; no attempt was made to adapt to the walls of the cavity. The fillings were made in a square cavity .085 by .085 of an inch. Twenty-four lots of gold, two grains each, were weighed out. Twelve of these lots were No. $\frac{1}{2}$ cylinder and twelve were tape. The tape was made by folding No. 4 gold until it was sixteen leaves thick and cutting to a convenient width for a cavity.

The same manufacturer's gold was used in each case. All was annealed on a sheet of mica over a small bunsen burner and made as uniform as possible. Two-hundred and eight blows were given each filling, after starting at bottom by hand pressure. Twenty-six pellets were set aside for malletting and the remaining four or five used for starting the filling. Eight blows were given each pellet. The tape gold was cut in strips and folded over and over upon itself. Eight fillings were made with each plugger, four using pellets and four using tape. The gold was not allowed to lap over edge of block. After making filling the surface of same was dressed down with a fine file and placed in a small box and labeled.

It is assumed that the cohesion made in these fillings would be shown by the density or specific gravity. The specific gravity of cast or hammered gold is 19.4 and the specific gravity of these fillings compared with the latter would show the amount of air space in each filling.

The specific gravity was determined by Prof. Fish of the Ohio State University. The method used was to get the volume into cubic millimeters and divide by weight in milligrams. Could not do it in water because amount of material was so small. Each dimension was taken by a micrometer. Being as careful as possible, by this means the specific gravity of different fillings were found as follows;

| | | | | | | |
|---|---|---|---|---|---|---|
| Fillings | made | with | sharply | serrated | pluggers | 13.9 |
| " | " | " | finely | " | " | 14.7 |
| " | " | " | smooth surface | " | " | 15.4 |

In the fillings made from pellets the average was found to be 14.5 and those made from tape 14.4. It will be seen by these that the density of fillings increased as the sharpness of pluggers decreased. The difference between the tape and pellet is so small that practically no advantage can be claimed by either.

In the next experiments a series of six fillings was made, using the same pluggers and the same methods except the fillings were made in round cavities 9-64 of an inch in diameter. The cavity into which the gold was packed was 11-64 of an inch deep, while the cavity which was used to hold the fillings, while being finished, was 7-64 of an inch deep. Two fillings were made with each plugger, one with tape and the other with rope. The ropes were cut into pieces about an inch long and were made by rolling about a quarter of a sheet of No. 4 foil. Eight grams of gold were used in each filling and 620 blows were struck. The mallet was set to a little harder blow. The specific gravity of these fillings when determined, were as follows;

| | | | | | | |
|---|---|---|---|---|---|---|
| Fillings | made | with | sharply | serrated | pluggers | 14.8 |
| " | " | " | finely | " | " | 15.4 |
| " | " | " | smooth surface | " | " | 16.4 |

The average specific gravity of those made from ropes was 15.55. The average of those made from tape was 15.56. The results with this series of fillings also shows that density increases as the serrations decrease.

In another series of six fillings, made in the same cavities and with No. 3-4 pellets, seven grains of gold were used in each and 910 blows struck each filling, or, fourteen blows to each pellet after the filling was started. Bayonet pluggers 3-64 of an inch in diameter with round face were used. The blow of the mallet was increased in force. The average in this series was; for the serrated point 12.83, for the smooth 15.43, showing a decided difference between the smooth and serrated pluggers. The results from all these experiments leads one to believe that serrated pluggers have no value as far as cohesion of gold is concerned. Cohesion is the molecular force acting at insensible distances between molecules of a substance, and the more smoothly and closely the surfaces or particles are brought together the stronger the cohesion. The real use of serrations is to keep the plugger from slipping, and from these experiments it would seem best if we are to have any at all, they should be very fine. The fact that gold will weld in a cold state is probably due to the lack of oxidation on the surface and its softness. Sharp serrations may be good when the gold is moist or its surface soiled; the pluggers cutting through the gold brings the fresh surfaces together.

In the beginning of my experiments I made my fillings and then rolled them out very thin. Made them in square cavities one by hand pressure with serrated pluggers and the other with smooth pluggers and mallet. The average density of the first was 13.1, that of the second 17.1. Very little difference could be seen after rolling because the pressure of the rolls seemed to make the condensation uniform. The same blow was used in each and about that ordinarily used in the mallet. The great increase of density of the filling indicates that there may be some principles worthy of consideration in regard to mallets. The size of a plugger point must be only large enough to keep it from penetrating the gold. The penetrating power decreases as the area of the point increases.

The next question which comes up is what kind of a blow is necessary for the best results? The momentum is equal to the product of the velocity and mass. When two masses come together the motion of one is imparted to the other in proportion to the momentum of each. When a ball is struck by a bat it has an amount of motion imparted to it in proportion to the weight and velocity of bat. The amount of work done is in proportion to the square of velocity. If the velocity of a body is increased the penetrating power is squared not doubled. The penetrating power of a moving power is equal to the square of the velocity. Applying this rule to the mallet at work in condensing gold, we should have a light mallet with a high velocity. It should be light so as to impart little of its own motion to the tooth and the velocity should be high to increase as much as possible the work at the point of the plugger or penetration. The superiority of the hand mallet is because the blow is struck at the end of a long handle, a lever of third class, which gives great swiftness of motion. Automatic mallets are not so good because of the friction of the plunger on the sides of the case, which impedes its velocity and prevents a sharp, quick blow. The most successful automatic mallets are those which lift the hammer freely from the plugger and return it with a sharp, quick impact. Those made on the plan of pushing or the inclined plane have failed. The elasticity in the mallet destroys or reduces by so much as it is elastic, the force of the blow by increasing the time of impact.

The reaction of the elastic substance reduces the energy by carrying the blow over a longer period of time, and this is a serious defect. If the hand mallet is used, it must be of considerable weight, because we can not get the velocity by the hand. Steel mallet is good because of its hardness and inelasticity, but it must be used with greater accuracy and skill so that its smooth surface may not change the direction of the force. For these reasons the lead-filled mallet is good. It does not slip and is not unduly elastic, So far as the blow is concerned, the electric mallet is the best we have. The blow of it is very rapid and the hammer of steel has no elasticity, and we get very little of its motion imparted to the tooth.

DISCUSSION.

Dr. H. L. Ambler: The experiments of the essayist show a great deal of perseverance and care and have been carried along in much the same lines as those of Dr. Black, and many of his conclusions are the same. However, Dr. Black used serrated pluggers and the hand mallet only. He also rested his steel cavity dyes on a rubber cushion, in this way getting as near as possible the natural conditions of the mouth. This the essayist, I suppose, did not do. In the experiments of Dr. Hawley he obtains results which show that fillings made with smooth pluggers have much higher specific gravity than those made with serrated pluggers, both sharp and fine. I do not know whether we all understand alike these terms sharp, fine and smooth. I can easily see how serrations of instruments may be sharp and deep at the same time. If they were so, I think he should have said so. They can also be fine and shallow, and the latter may be very fine or hlunt. I

would conclude from his statements that he undoubtedly means fine shallow serrations and sharp deep serrations. In the first series of experiments he was able to get fillings of much greater density with smooth pluggers than with serrated. Now this may be possible with some operators, those who are familiar with that style of plugger, but I venture to say there are many operators who will make just as dense, if not denser fillings with serrated pluggers. I do not think it possible to make a large contour filling with smooth pluggers. The smooth plugger wouid slip over the surface and not condense the gold, thus rendering the filling liable to break away. The doctor was also able to prove by his experiments that the form of gold made no difference in the density of the filling. However, I think with some operators the form of gold does make a great difference. Dr. Black tested this and found that the majority of operators were more successful with the tape form of gold, folding it over and over upon itself, and they did it with serrated pluggers. All these experiments proved nothing to me except that Dr. Hawley could do this—he could put more gold into those cavities with smooth pluggers than with serrated. The automatic plugger, as he used it, was a very good method for his purpose; but so far as we are concerned it is of no practical value, because the great majority of practitioners are to-day using the hand mallet. It seems strange, also, that no attempt was made to get tight margins in the fillings, because if he had intended to do so there would have been more gold in the cavity and fillings would have had greater specific gravity. He claims that cast and hammered gold have a specific gravity of 19.4. I think he copied that wrong. Dr. Black tested that matter over and over again; he made cast and hammered blocks of exactly the same dimensions, and then putting on stress he found that the cast block flowed five times as much as the hammered and that its specific gravity was less. The best filling that Dr. Black was able to make in a tooth had a specific gravity of 18.61; the majority of fillings made by other dentists which he tested were much below that. Dr. Black found that when the stress was put on the filling it immediately began to flow. At 100 pounds pressure there was no flow of the ordinary, well-made filling; at 150 pounds the gold changed shape and there was about 1 percent to 2 percent shortage. The question whether made with cohesive or soft foil made no difference. The standard filling of 19.4 specific gravity showed no flow under 150 pounds, but under 200 pounds it began to show some. Dr. Black had a number of dentists make gold fillings by use of hand mallet. On collecting them and placing under stress of 200 pounds pressure he found that there was 6 percent of shortage. He also found that in fillings made with corrugated gold foil there was 10 percent shortage. In fillings made with crumpled foil the average was 7 percent shortage. One of the best fillings made with hand mallet showed a shrinkage of 4 percent. I think the hand mallet is the best, and I like the suggestion of the essayist in regard to mallet, light mallet with high velocity. I use a mallet weighing two and a half ounces.

There are three different ways in which the hand mallet is used by operators. First, the plugger is placed on the gold, then struck by mallet; second, the plugger is pushed into place wherever the blow is to be struck, then struck with the mallet while pushing; third, the plugger is simply guided or held over the place where the blow is to be struck and then driven in with the mallet.

In reference to the serrations of instruments, I think that should be governed largely by the style of operation, thickness of gold used, and the kind of mallet employed.

Dr. J. B. Beauman: This paper is very important from my standpoint, although it may not be so from that of others. Of course we are all cranks, but some are bigger than others; and we all have hobbies—this is one of mine. I have been using the smooth plugger exclusively for over thirty years, and of course using them that long accounts for my strong inclination in that direction. I say if any man will lay aside serrated plugger for six months and use exclusively smooth, he will never go back to the serrated again. I ought to rip Dr. Ambler up the back, but do not wish to take take the time of the society. In this, as in many other things, it is necessary to understand certain fundamental principles. Gold, like all other substances, is composed of particles of matter called molecules, which are held together by that which man knows nothing about but which he calls molecular attraction. This principle is illustrated by cohesion etc. The principle of condensing gold with serrated instruments I can describe by a homely illustration. It is just like packing sand in a barrel with a pitchfork. The fine molecules of matter are chopped and divided up by the serrated instruments.

Dr. Ambler made the statement that it would not be possible to build up a good contour filling with smooth pluggers; he thought it would break off. It is just the reverse, however. Serrated pluggers disintegrate the gold in such a manner that it will not cohere. You can take pure gold and hammer it down until it is almost as hard as steel; the molecules are so welded one upon another that they can not yield any more, and there are no air-spaces between the molecules.

Hammered gold gives more resistance than cast. The reason is that the molecules are brought into closer contact, and I say it is with the smooth pluggers that this is better done. If you do not believe what I say, try it. You have a few instruments you prefer above all others; take off all serrations of these instruments and use them a short while, and, as I said before, you will never again go back to serrated pluggers. In reference to packing against the margins of a cavity, you are twice as apt to bruise the enamel rods with serrated points as with smooth.

New things are always coming up, which we try and cast aside because we really do not understand them, and we at once condemn, while later on we may take them up and find great merit in them. I remember when the rubber dam came in I thought it a great idea. I got some and commenced to use it, but would simply punch one hole to cover over several teeth. This, of course, made it worse than no rubber at all. I then threw it aside and claimed that the use of the rubber was no good. Later on I got the idea of punching a hole for each tooth; then I realized my great mistake. We do many things just as bad as that. We do not see it at first, but it becomes perfectly plain afterwards.

Dr. C. R. Butler: This paper shows that the essayist has taken a great deal of care in making his experiments. Yet so far as the main object in using gold, that for which we are all really here to learn, it does not amount to anything. All these experiments are made out of the mouth, simply packing in holes of a steel block, which is not the same as filling teeth in the mouth, it is not putting gold where it is to be utilized. The idea is to put in a filling which will stand the stress of mastification and preserve that tooth from all recurrence of decay.

Dr. J. R. Stephan: These experiments in steel dies I think are quite meritorious. To my mind Dr. Hawley is working in the right direction. The criticisms which have been offered in regard to condensing the gold along the margins, etc., which he has received here to-day will undoubtedly make him more careful in all his experiments in the future. It has been demonstrated long before that a small plugger with a rapidly repeated blow of the mallet is much more effective than a large plugger with a less rapid blow of equal strength. The idea that a smooth plugger will give us a harder filling than the serrated, is a point well worth recognizing, and these experiments have brought this fact out clearly. I hope Dr. Hawley will continue this line of experimentation and ascertain whether certain kinds of gold will not give us better results over others. If he will do so, I think we shall yet hear more new things of Dr. Hawley, as to how we may be able, in the mouth, to produce better operations using the proper instruments and forces which may be discovered in this way, just as was demonstrated by working out of the mouth, that small pluggers with rapidly repeating blows of the mallet were more effective than the large pluggers with a slower blow. The experiments of Dr. Black have lead many operatives who use a large plugger to use a much heavier blow than before in order to get the resisting qualities which are so necessary in a properly made filling.

Dr. Henry Barnes: I believe the object of Dr.

Hawley's paper was not to prove the correctness of his methods but to prove the results of his methods, and that he might receive such suggestions and criticisms upon it as would enable him to go on in the future and correct whatever errors he may have made. I think we should commend every action made in an advanced direction even if we do not agree with all the methods.

Dr. J. Taft: It seems to me the criticisms that have been offered are clearly applicable to the experiment. The full force of the argument was upon the amount of gold which could be put into a given cavity. The success of a filling does not depend so much upon this as it does upon perfect adaption to a cavity. This is especially so in the vicinity of the borders of the cavity, we must have absolute contact to exclude all foreign substances. This may be done without special reference to thorough condensation in the center of filling. It is not the best thought of the matter to see how much gold can be crowded into a given cavity; if we make thorough protection of the walls of a cavity, the center will take care of itself. The center of a gold filling may be comparatively loose and not as thoroughly condensed as possible, and yet be a good filling. I have no doubt that many times too much condensation is made in the center of a filling.

Dr. Mozart: I think the discussion has been altogether different than Dr. Hawley expected. He wanted to show how you can get a better density if you use smooth-pointed pluggers than you can if you use serrated pluggers; consequently this matter about other qualities in the making of a perfect filling should not be discussed.

Dr. Otto Arnold: I agree with the last speaker.

The proposition in the paper by Dr. Hawley is indeed very meritorious, showing lots of work, so far as the principle is concerned which he endeavored to demonstrate in the essay—the simple matter of density brought about by the use of different instruments. In reference to the smooth points he, of course, has advocated absolutely the use of only smooth points in contradistinction to what is known as serrated points, which are quite broad in extent. I think there is, like all questions of this sort, a middle ground; and I think the tendency of the general profession for many years has been toward the smooth points, as the serrations of the modern pluggers have been very shallow indeed, almost no serrations, merely a roughened surface. The advantage of the last named surface over polished, I think will be readily seen by all. Of course it depends a great deal upon the size and shape of the cavity; if it is very simple, with four walls, it might be filled with smooth pluggers, but in more labored operations in building up large contour fillings the disadvantage of polished points is to my mind quite apparent. There is likely to be a slipping of the plugger, which I fear may cause many times poor condensation. In an article written by Dr. Perry, of New York, in the *Cosmos*, he spoke of the preparations of fillings and applied a term which I think is more appropriate than most others I have heard, and that is "caulking." He compared it to the caulking of a vessel, perfect adaptation to exclude moisture being the essential feature. I would say, from my standpoint, that the modern plugger with very shallow serrations would approach more nearly my ideal for a plugger than that of a polished surface.

Dr. Hawley: I did not intend to cover the whole ground of manipulation of gold, in fact I did not intend to touch upon it at all. The only question that I did intend to cover was: What is the best kind of instrument to thoroughly condense gold filling material? There are some fundamental principles and some physical characteristics of gold to which we must adapt our practice. We can not adapt gold to our practice. We must adapt ourselves to the characteristics of gold. One would think from some of the discussion offered that we could put gold anywhere we liked.

There has been some misapprehension in regard to the methods of making my experiments, which was, perhaps, my fault. A good many details I did not mention. In regard to the position of the block upon the bench, this steel cavity block was placed upon the hard bench with simply a napkin underneath it. Any other surface is not uniform, and I wish to make each case uniform, The object of Dr. Black's experiments was not to test the advantage of different plugger points at all, but simply to answer the question, Are we making gold fillings to-day dense enough to resist the crushing force of the jaw? For that reason he passed his cavity block around among something like thirty dentists and allowed them to use any method, whatever mallets or kinds of gold they wished. The results showed that fillings made to-day by very good dentists are lamentably lacking in strength. As a result of his experiments the question came to me how to make these fillings stronger, and I took up this simple question.

In regard to the serrations used, the serrations in the first set of pluggers were very nearly those that are ordinarily used, point being small (1-32 x 1-64), with deep serrations. The second set used were finely serrated, and the third I myself made perfectly smooth.

I selected the pluggers from the stock sold in all the dental depots and just as are generally used by the dentists.

In regard to the mallet, I stated in the paper I used that in which I could determine and keep at a fixed blow. I prefer a hand mallet, but it is not practical in these experiments at all ; must have the same blow at all times, the force of blow must be the same, and amount of gold used in each filling must be the same.

In reference to the adaption to the margin, I did not mean that I was careless about it and in fact I did not care to get perfect margins. To get them perfect it would have been necessary to direct the blow straight against the margin and this would cause a difference in the fillings ; so I got as close adaption as possible by striking as nearly at right angles to the bottom of the cavity as I could.

In regard to the starting of a filling, "Why did I not start the fillings with mallet?" I do not know of any method by which a filling can be started in the bottom of a square steel cavity with a mallet ; it is necessary to have at first a mass of gold to retain its position. After placing just enough to get that supporting action, I then commenced the work with the mallet. In regard to the determination of the specific gravity of a hammered and cast gold there is little difference between the two, cast gold is 19.3 and hammered gold is 19.5 and we took a medium of 19.4. I am aware of course that the determination of the density does not settle the question of cohesion, it is possible to have a very dense filling and not have cohesion as Dr. Black showed in his experiments,

Dr. Butler, if he will pardon me, is mistaken in regard to experiments out of the mouth. If a dense filling is valuable in the mouth it certainly is important to determine how this can be made out of the mouth, and we can determine that just as well in a steel block as in anything else. The question about adaptation of the filling to the margins has not been taken up in these experiments at all. A filling to be perfect must be thoroughly condensed and certainly close the cavity; Dr. Beauman has used smooth points for thirty years, and if there is not a cohesion in his gold he would certainly have found it out long before this time. And if smooth pluggers bring about cohesion, it will certainly carry the gold over the border and adapt it to the edge and not fracture the enamel rod.

I am greatly disappointed in one respect as to the forms of gold. Before I commenced these experiments I believed that the tape gold made a denser filling, but all these experiments seemed to show I was not warranted in so thinking. Of course these few experiments, merely thirty gold fillings, are not sufficient to thoroughly determine this point.

In conclusion, I do not wish to advocate one way or another the practice of using serrated or smooth points, but what I wanted to bring before the Society and what I want to determine, is, when to use serrated and when to use smooth; and what is the comparative value of serrated points; are they valuable for condensing gold, or merely to keep the plugger from slipping?

